# Pioneering studies on monogenic central precocious puberty

**DOI:** 10.20945/2359-3997000000164

**Published:** 2019-08-14

**Authors:** Ana Pinheiro Machado Canton, Carlos Eduardo Seraphim, Vinicius Nahime Brito, Ana Claudia Latronico

**Affiliations:** 1 Universidade de São Paulo Hospital das Clínicas Faculdade de Medicina Universidade de São Paulo São Paulo SP Brasil Unidade de Endocrinologia do Desenvolvimento, Laboratório de Hormônios e Genética Molecular LIM/42 do Hospital das Clínicas, Disciplina de Endocrinologia da Faculdade de Medicina da Universidade de São Paulo, São Paulo, SP, Brasil

**Keywords:** Central precocious puberty, genetics, MKRN3, DLK1

## Abstract

Pubertal timing in humans is determined by complex interactions including hormonal, metabolic, environmental, ethnic, and genetic factors. Central precocious puberty (CPP) is defined as the premature reactivation of the hypothalamic-pituitary-gonadal axis, starting before the ages of 8 and 9 years in girls and boys, respectively; familial CPP is defined by the occurrence of CPP in two or more family members. Pioneering studies have evidenced the participation of genetic factors in pubertal timing, mainly identifying genetic causes of CPP in sporadic and familial cases. In this context, rare activating mutations were identified in genes of the kisspeptin excitatory pathway (*KISS1R* and *KISS1* mutations). More recently, loss-of-function mutations in two imprinted genes (*MKRN3* and *DLK1*) have been identified as important causes of familial CPP, describing novel players in the modulation of the hypothalamic-pituitary-gonadal axis in physiological and pathological conditions. *MKRN3* mutations are the most common cause of familial CPP, and patients with *MKRN3* mutations present clinical features indistinguishable from idiopathic CPP. Meanwhile, adult patients with *DLK1* mutations present high frequency of metabolic alterations (overweight/obesity, early onset type 2 diabetes and hyperlipidemia), indicating that *DLK1* may be a novel link between reproduction and metabolism. Arch Endocrinol Metab. 2019;63(4):438-44

## INTRODUCTION

Pubertal development results from the re-emergence of pulsatile hypothalamic gonadotropin-releasing hormone (GnRH) secretion, which is coordinated by a partially clarified mechanism involving inhibitory, stimulatory, and permissive factors acting upstream of GnRH neurons (1). Moreover, pubertal timing in humans is determined by complex interactions of different influences, such as metabolic, environmental, ethnic, and genetic factors (2). Genereally, it occurs between the ages of 8 and 13 in girls; and 9 and 14 in boys (2). Therefore, puberty is considered precocious if it starts prior to the ages of 8 and 9 in girls and boys, respectively (2).

The involvement of genetic factors in puberty control has been indicated by several evidences, such as similar age at menarche between mother and daughter, as well as among individuals of the same ethnic group; higher correlation in pubertal timing between monozygotic twins than between dizygotic twins or sibling pairs; and large-scale genome-wide association studies (1,3). It is estimated that 60-80% of the variation in pubertal timing is due to genetic factors (1). Familial precocious puberty is typically defined by the occurrence of more than one affected family member (4).

Central precocious puberty (CPP) results from the premature activation of the hypothalamic-pituitary-gonadal axis (HPG) and is considered “idiopathic” or of unknown causes, when congenital or acquired lesions in the central nervous system and monogenic defects are ruled out (2). Idiopathic CPP frequency varies according to population registries. In American girls, the incidence was estimated to be 1 in 5,000 to 10,000; however, in Danish girls, the prevalence was 1 in 500 (5). Regardless of the cohort, the prevalence of CPP is sexually dimorphic, being higher in girls than in boys (2). Up to now, Brazilian registries regarding population frequency of idiopathic CPP are unavailable. The occurrence of familial cases of CPP, not always detectable through anamnesis, fortifies the genetic basis of this disorder. De Vries and cols. (4) studied a large cohort of children with idiopathic CPP and identified a familial component in approximately 27% of them. Notably, inheritance was described in both maternal and paternal transmission in an autosomal dominant inheritance pattern with incomplete penetrance.

The diagnosis of CPP is based on physical exam findings indicating progressive puberty and on laboratory evaluation confirming central HPG axis activation, mainly through basal or GnRH-stimulated luteinizing hormone (LH) levels. After biochemical confirmation of CPP, a magnetic resonance imaging of the central nervous system must be performed in all patients to exclude anatomical abnormalities (2).

**Figure 1:**
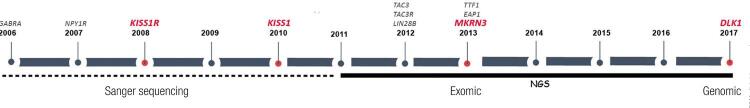
Timeline of the pioneering studies on monogenic central precocious puberty with the main participation of the Brazilian research group of pubertal disorders studies from the Developmental Endocrinology Unit at the São Paulo University Medical School.

In recent years, genetic factors that regulate the HPG axis and modulate pubertal timing have been partially elucidated, primarily enabled by more sensitive and powerful molecular biology tools and epidemiological studies, such as large-scale genome-wide association studies, genomic microarray, whole exome sequencing, and, more recently, whole genome sequencing. This diagnostic advancement is revealing genetic causes of CPP involving novel players that modulates the HPG axis in physiological and pathological conditions (1) (Table 1). In this context, the Brazilian research group of pubertal disorders studies from the Developmental Endocrinology Unit at the São Paulo University Medical School has conducted pioneering studies with a prosperous collaboration from American researchers (Figure 1). Herein, we revise the current known genetic causes of CPP, emphasizing on monogenic causes.

**Table 1:** Genetic causes of central precocious puberty

Monogenic causes
1.Gain-of-function (activating) mutations in *KISS1R* and *KISS1* genes (kisspeptin pathway genes)
2.Loss-of-function (inactivating) mutations in *MKRN3* gene
3.Loss-of-function (inactivating) mutations in *DLK1* gene

**Chromosomal abnormalities**

1.Temple syndrome: maternal uniparental disomy, epimutation or paternal deletion at chromosome 14q32.2
2.Silver-Russell syndrome: maternal uniparental disomy of chromosome 7
3.Williams-Beuren syndrome: 7q11.23 deletion
4.Prader-Willi syndrome: maternal uniparental disomy or paternal deletion at 15q11-q13
5.Rare cases of distinct copy number variants: 1p36 deletion, 9p distal deletion, 9q34.3 duplication, Xp22.33 deletions, Xp11.23-p11.22 duplication, and *CDKL5* gene deletion

### Monogenic causes of central precocious puberty (Table 2)

#### 1. Activating mutations in genes KISS1 and KISS1R leading to sporadic CPP

**Table 2:** Genetic and phenotypic characteristics of reported patients with monogenic causes of central precocious puberty

Gene	Patients reported	Genetic defects (mutations)	Inheritance	Pubertal development, yr		Other clinical features	Ref.

				Puberty onset	Menarche (untreated)		
*KISS1R*	1 (F)	Gain of-function missense	-	Thelarche since birth Progression at 7	-	-	(6)
*KISS1*	1 (M)	Gain-of-function missense	Autosomal dominant with incomplete sex-dependent penetrance	1	-	-	(7)
*MKRN3*	89 (76F:13M)	Loss-of-function: 1.Frameshift 2.Nonsense 3.Missense 4.Promoter deletion	Autosomal dominant with paternal expression	Female 6.0 (3.0 to 7.8) Male 8.0 (5.9 to 9.0)	-	Indistinguishable from idiopathic CPP	(8,15,20)
*DLK1*	10 (F)	Loss-of-function: 1.Deletion 2.Frameshift	Autosomal dominant with paternal expression	5.5 (4.6 to 7.0)	7.8 (7.0 to 9.0)	Overweight/obesity Early type 2 diabetes Hyperlipidemia Polycystic ovary syndrome	(22,23)

In 2008, the first monogenic defect in a patient with CPP was identified: a heterozygous activating mutation (p.Arg386Pro) of *KISS1R* gene, also called *GPR54*, which encodes the kisspeptin receptor (6). By biological plausibility, activating mutations of the kisspeptin pathway were expected to cause premature activation of the HPG axis, thus leading to CPP. This mutation was identified in an adopted girl presenting progressive thelarche since birth followed by accelerated growth, skeletal maturation, and development of progressive secondary sexual characters at age 7. *In vitro* studies showed that the mutation led to prolonged activation of intracellular signaling pathways in response to kisspeptin (2,6).

Parallel with the first kisspeptin pathway activating defect discovery, Silveira and cols. (7) identified an activating heterozygous mutation in the kisspeptin gene (*KISS1*) (p.Pro74Ser). This rare variant was identified in a boy who developed very early sporadic CPP at age 1. His mother and maternal grandmother were carriers of the same mutation, despite normal pubertal development, thus suggesting an incomplete sex-dependent penetrance of the phenotype*. In vitro* studies identified that the p.Pro74Ser mutation had a greater capacity to stimulate signal transduction than the wild type, leading to greater kisspeptin bioavailability (2,7).

Both activating mutations of *KISS1R* and *KISS1* genes associated to CPP cases contributed to the elucidation of the role of kisspeptin pathway in pubertal control in physiological conditions. However, no other CPP cases with activating mutations in the kisspeptin pathway have been reported since then, suggesting these are very rare molecular mechanisms of CPP.

#### 2. Inactivating mutations in imprinted gene MKRN3 leading to familial CPP

With the increasingly available techniques of next generation sequencing, the investigation of the genetics of CPP has further progressed. Abreu and cols*.* (8) first described the role of makorin RING-finger 3 (*MKRN3)* in the pathogenesis of CPP in 2013. Whole exome sequencing analysis of 32 patients with CPP (27 girls and 5 boys) identified 15 individuals (8 girls and 7 boys) from 5 families with CPP who harbored *MKRN3* inactivating mutations. Four families had frameshift mutations, while the fifth family had a missense variant that was predicted to be deleterious and causes loss-of-function (8).

**Figure 2:**
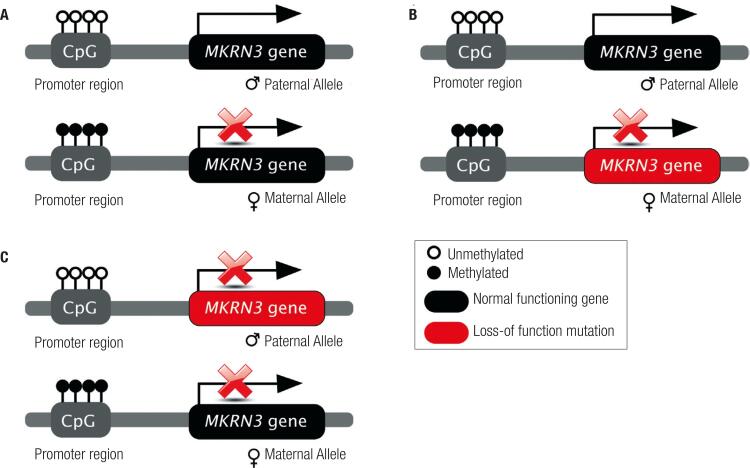
(A) Normal pattern of imprinting of *MKRN3* gene, with silencing of the maternal allele (by methylation of its promoter region) and monoallelic expression of the paternal allele. (B) The genotype of a patient who inherited a maternal loss-of-function mutation. The patient will not present central precocious puberty, because the paternal allele is normally expressed. (C) The genotype of a patient who inherited a paternal loss-of-function mutation. The patient will present central precocious puberty because both alleles are inactive: the paternal is mutated and the maternal is silenced.

*MKRN3* is an intronless gene, located at the imprinted locus 15q11-q13, the critical region of Prader-Willi and Angelman syndromes (9,10). Genomic imprinting is a physiological process of gene silencing, which is defined as the monoallelic expression of a gene according to its parental origin. Therefore imprinted genes are silenced (imprinted) in one parental allele and expressed only from the other (11). DNA methylation usually occurs in cytosine nucleotides located within CpG islands at promoter or intergenic regions, resulting in transcriptional silencing (9,11). *MKRN3* presents maternal imprinting (maternal allele silencing), therefore, patients present CPP only when they inherit the mutated allele from their fathers (Figure 2) (8,9). The *MKRN3* gene encodes a 507-amino acid protein with a RING (C^3^HC^4^) zing finger motif and multiple C3H zinc finger motifs (9). The gene is expressed ubiquitously as a 3-kb transcript, with a 5-prime CpG island region that is methylated on the maternal allele. *MKRN3* is associated with protein ubiquitination, a process in which a ubiquitin is attached to a protein, and in turn tags it for movement to the proteasome, where it is then degraded (9).

In their initial report, Abreu and cols. (8) performed real-time PCR in the arcuate nucleus of mice. In both sexes, the *Mkrn3* mRNA levels were highest on postnatal days 10 and 12 and declined subsequently, reaching their nadir by days 18-22, in the same period that the *Kiss1* and *Tac2* expression were shown to increase, marking the onset of puberty (8). Therefore, it was concluded that *MKRN3* had an inhibitory role on pubertal onset, and that its function loss would favor the premature stimulation of GnRH secretion and puberty development. This finding was corroborated by a study in Danish girls, in which circulating serum levels of MKRN3 where shown to decrease by 15% preceding pubertal onset; furthermore, MKRN3 levels were lower in early maturing girls when compared witg age-matched prepubertal girls (12). However, up to now, a cutoff value that accurately predicts puberty has not been established (13). Furthermore, the exact mechanism through which *MKRN3* expression modulates GnRH secretion and puberty has not been fully elucidated, although a few factors have been recently described (14).

Subsequently, *MKRN3* mutations were investigated in a large cohort comprising patients from three Brazilian university hospitals. Macedo and cols*.* (15) studied 215 patients with apparently sporadic CPP, identifying eight new cases of CPP caused by *MKRN3* loss-of-function mutations. In another study, Bessa and cols.*.* (16) reported *MKRN3* mutations in 8 out of 20 boys with apparently idiopathic CPP, reporting a high frequency of *MKRN3* mutations in male with CPP previously classified as idiopathic. Further studies performed in centers from different countries, as well as a recent systematic review, confirmed that defects in *MKRN3* are the most common cause of genetic CPP, with prevalence ranging from 33 to 46% in familial cases (15,17), and 0.4 to 5% in sporadic cases (16,18-20).

Further evaluation through genome sequencing of 115 patients aiming to investigate possible pathogenic variants in noncoding regions revealed a rare heterozygous deletion in the proximal promoter region of *MKRN3* in a girl with CPP (21). *In silico* analysis predicted that this variant would lead to a loss of a downstream responsive element antagonist modulator (DREAM) binding site, which would lead to a decrease in *MKRN3* expression; this finding was confirmed with the *in vitro* reduction of the promoter activity (21).

Currently, more than 30 different loss-of-function mutations of *MKRN3* have been reported since the first report by Abreu and cols. (20) (Table 1). Notably, a large proportion of these mutations were frameshift affecting the amino-terminal region of the protein that is codified by nucleotides between 476-482, which consists of a rich poly C site, suggesting that this area represents a hotspot for inactivating mutations in CPP.

The clinical features of CPP caused by *MKRN3* inactivating mutations are indistinguishable from other idiopathic CPP. The median age of puberty onset is 6 in girls and 8 in boys, with the youngest reported case being a 3-year-old girl with Tanner stage 3 breast development and advanced bone age. Up to now, remarkable clinical or hormonal features distinguishing patients with CPP caused by *MKRN3* mutations are not identified (8). Moreover, the response to treatment with GnRH agonists appears to be adequate, as outlined by Macedo and cols*.* (15).

#### 3. Inactivating mutations in imprinted gene DLK1 leading to familial CPP

In 2017, Dauber and cols. (22) studied a three-generation Brazilian family with five affected members (two sisters, two paternal half-sisters, and their paternal grandmother) using linkage analysis followed by whole-genome sequencing. The children’s fathers had normal pubertal timing, suggesting an autosomal dominant inheritance either with incomplete penetrance or an imprinting mechanism. A complex defect in the Delta-like 1 homolog (*DLK1*) imprinted gene was identified (14-kb heterozygous deletion that encompassed the first exon of the gene). Moreover, sequencing analysis showed also a 269-bp duplication from intron 3 of *DLK1*, segregating with the deletion (22). *DLK1* is an imprinted paternally expressed gene, meaning that carriers of *DLK1* defects only manifest the phenotype if the defect is inherited from their fathers, similar to *MKRN3* affected families.

Subsequently, three novel families with *DLK1* loss-of-function mutations with paternal expression (phenotype was expressed only when the mutation was inherited from the father) were described by Gomes and cols. (23), who performed *DLK1* sequencing analysis in 60 patients with CPP or history of precocious menarche. Three distinct frameshift mutations in the exon 5 of *DLK1* were identified in five patients from three unrelated families (p.Gly199Alafs*11, p.Val271Cysfs*14 and p.Pro160Leufs*50) (23).

DLK1, also called preadipocyte factor 1 (Pref-1), is a transmembrane protein containing epidermal growth factor-like repeats in its extracellular domain (22). It is a noncanonical ligand in the Delta-Notch signaling pathway, known to play a role in several cell types differentiation, mostly in inhibiting adipocyte differentiation (22,24). DLK1 has a wide expression in fetal life; however, in postnatal life, the expression decreases, except in endocrine glands (mainly in the adrenals, the pituitary and the ovaries) (22,24). A hypothalamic function of DLK1 has been suggested by evidence of its expression also in several hypothalamic nuclei (24).

Human *DLK1* is a paternally expressed gene located at chromosome 14q32.2, a region encompassing a cluster of imprinted genes (25). Maternal uniparental disomy, epimutations and paternal deletions at chromosome 14q32.2 lead to expression loss of the paternally expressed genes of this region, including *DLK1*. These molecular abnormalities are associated to Temple syndrome, an imprinting disorder mainly characterized by pre- and postnatal growth failure, hypotonia, small hands and feet, CPP, and overweight/obesity after infancy (26-28).

To investigate the effect of mutations on DLK1 production, serum DLK1 levels were measured in the affected family members (mutated *DLK1* patients) and in controls, using an available soluble DLK1 enzyme-linked immunosorbent assay (ELISA) (22,23). Serum DLK1 concentrations were undetectable in all tested affected individuals. Moreover, Abi Habib and cols. (11) identified undetectable levels of serum DLK1 in Temple syndrome patients with epimutations and deletions at chromosome 14q32.2. Taken together, these data indicate that serum DLK1 measurement may be a potential screening method for investigating DLK1 deficiency in affected children.

Interestingly, patients with *DLK1* mutations presented higher metabolic abnormalities when compared with patients with patients with idiopathic CPP as demonstrated by Gomes and cols*.* (23), thus suggesting that this antiadipogenic factor represents a novel link between reproduction and metabolism. The more prevalent features at adulthood were central obesity, early onset glucose intolerance/type 2 diabetes, and hyperlipidemia. These metabolic alterations were similar to the phenotype of *Dlk1* deficiency previously described in a null mouse model (23). In addition, patients with Temple syndrome may develop obesity, early onset type 2 diabetes and hyperlipidemia as young adults (26-28).

Another clinical aspect demonstrated by Gomes and cols. (23) was the presence of polycystic ovarian syndrome (PCOS) in two out of 10 adult women evaluated with *DLK1* mutations. It is postulated that a neuroendocrine component may participate in PCOS physiopathology with increased GnRH pulse frequency leading to increased LH production over FSH production. It is well established that girls with CPP may present an abnormality in GnRH pulses. The authors suggested that this phenomenon, as well as the poor metabolic profile, could link both conditions (23).

In addition, the DLK1 protein function has been studied in distinct metabolic settings. It has been proposed that DLK1 may alter the metabolic mode of the organism from lipid storage to peripheral lipid oxidation, protecting the organism from hepatic steatosis (29). Interestingly, the exogenous administration of DLK1 in mice reduced hepatic steatosis and hyperglycemia via AMPK activation in the liver (30). Moreover, in pregnancy, it has been shown that elevated DLK1 circulating levels are required to the increased maternal fatty acid metabolism. In a study evaluating late gestation, Cleaton and cols*.* (31) demonstrated that maternal levels of DLK1 were fetus-derived and could be used as a marker of complicated fetal growth restriction.

## Chromosomal abnormalities

A few studies have reported rare cases of patients with idiopathic CPP associated with complex phenotypes, primarily related to clinical syndromes or chromosomal abnormalities (1,32). To date, CPP has been reported as part of the phenotypic spectrum of the following genetic syndromes: 1) Temple syndrome (maternal uniparental disomy or epimutation 14q32.2) (90% of cases) (27,28); 2) Silver-Russell syndrome (maternal uniparental disomy of chromosome 7) (up to 25% of case) (2,33) 3) Williams-Beuren syndrome (7q11.23 deletion) (10-18% of cases) (32); 4) Prader-Willi syndrome (15q^11^-q^13^ paternal deletion) (4-10% of cases) (1). Additionally, studies have been reported regarding patients with idiopathic CPP associated with distinct copy number variants, such as 1p36 deletion, 9p distal deletion, 9q34.3 duplication (including *NOTCH* gene), Xp11.23-p11.22 duplication, and *CDKL5* gene deletion (1). Notably, Temple syndrome, Silver-Russell syndrome, and Prader-Willi syndrome are imprinting disorders (11).

**Figure 3:**
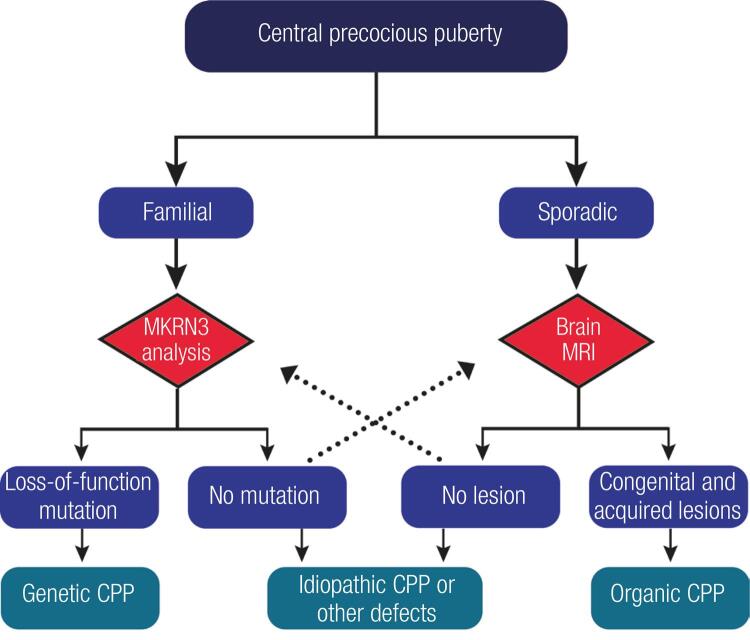
Flow chart for investigation of familial central precocious puberty.

In conclusion, central precocious puberty is considered idiopathic in most children, particularly in girls. This scenario may change with the identification of genetic causes underlying CPP. Recent identifications of *MKRN3* and *DLK1* mutations in familial CPP established the relevance of genetic factors in the physiopathology of this condition. In addition, some studies have emphasized the association of CPP with rare cases of genetic and epigenetic defects characterized by clinical syndromes or complex phenotypes. In this context, the selection of patients with CPP for genetic testing should consider the family history and segregation (family members with similar phenotype), and the presence of other clinical features (Figure 3). Genetic testing has now become a more routine clinical practice, thus allowing a greater number of patients to be properly tested.

It is noteworthy that patients with genetic causes of CPP have been described as presenting adequate clinical and laboratory responses to long-acting GnRH analogs (the gold standard treatment), similar to patients with idiopathic CPP. Nevertheless, careful monitoring for reproductive, metabolic, and general health related outcomes should be considered during management of patients with CPP with genetic conditions, including an extended follow-up until adulthood.

## References

[1] Macedo DB, Silveira LF, Bessa DS, Brito VN, Latronico AC. Sexual Precocity – Genetic Bases of Central Precocious Puberty and Autonomous Gonadal Activation. Endocr Dev. 2016;29:50-71. 10.1159/000438874 26680572

[2] Latronico AC, Brito VN, Carel JC. Causes, diagnosis, and treatment of central precocious puberty. Lancet Diabetes Endocrinol. 2016;4(3):265-74. 10.1016/S2213-8587(15)00380-0 26852255

[3] Day FR, Thompson DJ, Helgason H, Chasman DI, Finucane H, Sulem P, et al. Genomic analyses identify hundreds of variants associated with age at menarche and support a role for puberty timing in cancer risk. Nat Genet. 2017;49(6):834-41. 10.1038/ng.3841 PMC5841952 28436984

[4] de Vries L, Kauschansky A, Shohat M, Phillip M. Familial central precocious puberty suggests autosomal dominant inheritance. J Clin Endocrinol Metab. 2004;89(4):1794-800. 10.1210/jc.2003-030361 15070947

[5] Teilmann G, Pedersen CB, Jensen TK, Skakkebaek NE, Juul A. Prevalence and incidence of precocious pubertal development in Denmark: an epidemiologic study based on national registries. Pediatrics. 2005;116(6):1323-8. 10.1542/peds.2005-0012 16322154

[6] Teles MG, Bianco SD, Brito VN, Trarbach EB, Kuohung W, Xu S, et al. A GPR54-activating mutation in a patient with central precocious puberty. N Engl J Med. 2008;358(7):709-15. 10.1056/NEJMoa073443 PMC2859966 18272894

[7] Silveira LG, Noel SD, Silveira-Neto AP, Abreu AP, Brito VN, Santos MG, et al. Mutations of the KISS1 gene in disorders of puberty. J Clin Endocrinol Metab. 2010;95(5):2276-80. 10.1210/jc.2009-2421 PMC2869552 20237166

[8] Abreu AP, Dauber A, Macedo DB, Noel SD, Brito VN, Gill JC, et al. Central precocious puberty caused by mutations in the imprinted gene MKRN3. N Engl J Med. 2013;368(26):2467-75. 10.1056/NEJMoa1302160 PMC3808195 23738509

[9] Jong MT, Gray TA, Ji Y, Glenn CC, Saitoh S, Driscoll DJ, et al. A novel imprinted gene, encoding a RING zinc-finger protein, and overlapping antisense transcript in the Prader-Willi syndrome critical region. Hum Mol Genet. 1999;8(5):783-93. 10.1093/hmg/8.5.783 10196367

[10] Nicholls RD, Saitoh S, Horsthemke B. Imprinting in Prader-Willi and Angelman syndromes. Trends Genet. 1998;14(5):194-200. 10.1016/s0168-9525(98)01432-2 9613204

[11] Abi Habib W, Brioude F, Azzi S, Rossignol S, Linglart A, Sobrier ML, et al. Transcriptional profiling at the DLK1/MEG3 domain explains clinical overlap between imprinting disorders. Sci Adv. 2019;5(2):eaau9425. 10.1126/sciadv.aau9425 PMC6382400 30801013

[12] Hagen CP, Sørensen K, Mieritz MG, Johannsen TH, Almstrup K, Juul A. Circulating MKRN3 levels decline prior to pubertal onset and through puberty: a longitudinal study of healthy girls. J Clin Endocrinol Metab. 2015;100(5):1920-6. 10.1210/jc.2014-4462 25695892

[13] Busch AS, Hagen CP, Almstrup K, Juul A. Circulating MKRN3 Levels Decline During Puberty in Healthy Boys. J Clin Endocrinol Metab. 2016;101(6):2588-93. 10.1210/jc.2016-1488 27057785

[14] Yellapragada V, Liu X, Lund C, Känsäkoski J, Pulli K, Vuoristo S, et al. MKRN3 Interacts With Several Proteins Implicated in Puberty Timing but Does Not Influence. Front Endocrinol (Lausanne). 2019;10:48. 10.3389/fendo.2019.00048 PMC6375840 30800097

[15] Macedo DB, Abreu AP, Reis AC, Montenegro LR, Dauber A, Beneduzzi D, et al. Central precocious puberty that appears to be sporadic caused by paternally inherited mutations in the imprinted gene makorin ring finger 3. J Clin Endocrinol Metab. 2014;99(6):E1097-103. 10.1210/jc.2013-3126 PMC4037732 24628548

[16] Bessa DS, Macedo DB, Brito VN, França MM, Montenegro LR, Cunha-Silva M, et al. High Frequency of MKRN3 Mutations in Male Central Precocious Puberty Previously Classified as Idiopathic. Neuroendocrinology. 2017;105(1):17-25. 10.1159/000446963 PMC5195904 27225315

[17] Simon D, Ba I, Mekhail N, Ecosse E, Paulsen A, Zenaty D, et al. Mutations in the maternally imprinted gene MKRN3 are common in familial central precocious puberty. Eur J Endocrinol. 2016;174(1):1-8. 10.1530/EJE-15-0488 26431553

[18] Lee HS, Jin HS, Shim YS, Jeong HR, Kwon E, Choi V, et al. Low Frequency of MKRN3 Mutations in Central Precocious Puberty Among Korean Girls. Horm Metab Res. 2016;48(2):118-22. 10.1055/s-0035-1548938 25938887

[19] Ortiz-Cabrera NV, Riveiro-Álvarez R, López-Martínez M, Pérez-Segura P, Aragón-Gómez I, Trujillo-Tiebas MJ, et al. Clinical Exome Sequencing Reveals MKRN3 Pathogenic Variants in Familial and Nonfamilial Idiopathic Central Precocious Puberty. Horm Res Paediatr. 2017;87(2):88-94. 10.1159/000453262 27931036

[20] Valadares LP, Meireles CG, De Toledo IP, Santarem de Oliveira R, Gonçalves de Castro LC, Abreu AP, et al. MKRN3 Mutations in Central Precocious Puberty: A Systematic Review and Meta-Analysis. J Endocr Soc. 2019;3(5):979-95. 10.1210/js.2019-00041 PMC6483926 31041429

[21] Macedo DB, França MM, Montenegro LR, Cunha-Silva M, Best DS, Abreu AP, et al. Central Precocious Puberty Caused by a Heterozygous Deletion in the MKRN3 Promoter Region. Neuroendocrinology. 2018;107(2):127-32. 10.1159/000490059 PMC6363361 29763903

[22] Dauber A, Cunha-Silva M, Macedo DB, Brito VN, Abreu AP, Roberts SA, et al. Paternally Inherited DLK1 Deletion Associated With Familial Central Precocious Puberty. J Clin Endocrinol Metab. 2017;102(5):1557-67. 10.1210/jc.2016-3677 PMC5443333 28324015

[23] Gomes LG, Cunha-Silva M, Crespo RP, Ramos CO, Montenegro LR, Canton A, et al. DLK1 is a novel link between reproduction and metabolism. J Clin Endocrinol Metab. 2019;104(6):2112-2120. 10.1210/jc.2018-02010 30462238

[24] Villanueva C, Jacquier S, de Roux N. DLK1 is a somato-dendritic protein expressed in hypothalamic arginine-vasopressin and oxytocin neurons. PLoS One. 2012;7(4):e36134. 10.1371/journal.pone.0036134 PMC3338567 22563444

[25] Schmidt JV, Matteson PG, Jones BK, Guan XJ, Tilghman SM. The Dlk1 and Gtl2 genes are linked and reciprocally imprinted. Genes Dev. 2000;14(16):1997-2002. PMC316857 10950864

[26] Ioannides Y, Lokulo-Sodipe K, Mackay DJ, Davies JH, Temple IK. Temple syndrome: improving the recognition of an underdiagnosed chromosome 14 imprinting disorder: an analysis of 51 published cases. J Med Genet. 2014;51(8):495-501. 10.1136/jmedgenet-2014-102396 24891339

[27] Kagami M, Nagasaki K, Kosaki R, Horikawa R, Naiki Y, Saitoh S, et al. Temple syndrome: comprehensive molecular and clinical findings in 32 Japanese patients. Genet Med. 2017;19(12):1356-66. 10.1038/gim.2017.53 PMC5729347 28640239

[28] Geoffron S, Abi Habib W, Chantot-Bastaraud S, Dubern B, Steunou V, Azzi S, et al. Chromosome 14q32.2 Imprinted Region Disruption as an Alternative Molecular Diagnosis of Silver-Russell Syndrome. J Clin Endocrinol Metab. 2018;103(7):2436-46. 10.1210/jc.2017-02152 29659920

[29] Charalambous M, Da Rocha ST, Radford EJ, Medina-Gomez G, Curran S, Pinnock SB, et al. DLK1/PREF1 regulates nutrient metabolism and protects from steatosis. Proc Natl Acad Sci U S A. 2014;111(45):16088-93. 10.1073/pnas.1406119111 PMC4234615 25349437

[30] Lee YH, Yun MR, Kim HM, Jeon BH, Park BC, Lee BW, et al. Exogenous administration of DLK1 ameliorates hepatic steatosis and regulates gluconeogenesis via activation of AMPK. Int J Obes (Lond). 2016;40(2):356-65. 10.1038/ijo.2015.173 26315841

[31] Cleaton MA, Dent CL, Howard M, Corish JA, Gutteridge I, Sovio U, et al. Fetus-derived DLK1 is required for maternal metabolic adaptations to pregnancy and is associated with fetal growth restriction. Nat Genet. 2016;48(12):1473-80. 10.1038/ng.3699 PMC5373434 27776119

[32] Canton A, Brito V, Montenegro L, Ramos C, Macedo D, Bessa D, et al. Clinical and Genetic Features of Central Precocious Puberty Associated with Complex Phenotypes. Horm Res Paediatr. 2018:98-9.

[33] Canton APM, Harbison M, Abi Habib W, Salem J, Blaise A, Geoffron S, et al. Clinical and molecular analysis of pubertal control in a cohort of Silver-Russell and Temple syndrome patients. Horm Res Paediatr. 2017:9.

